# Comparative Physiological and Gene Expression Analyses Reveal Mechanisms Involved in Maintaining Photosynthesis Capacity, Alleviating Ion Toxicity and Oxidative Stress of Kentucky Bluegrass under NaCl Treatment

**DOI:** 10.3390/plants13152107

**Published:** 2024-07-30

**Authors:** Rong Wang, Shi-Jie Yan, Chao Liu, Huan Guo, Yan-Nong Cui

**Affiliations:** College of Grassland Agriculture, Northwest A&F University, Yangling 712100, China; wr00142023@163.com (R.W.); yjay@nwafu.edu.cn (S.-J.Y.); liuchao3314463317@163.com (C.L.)

**Keywords:** salt stress, photosynthesis, Na^+^ transport, ROS, turfgrass

## Abstract

Kentucky bluegrass (*Poa pratensis* L.), a widely used cool-season turfgrass, shows a high sensitivity to soil salinity. Clarifying the adaptative mechanisms of Kentucky bluegrass that serve to improve its salt tolerance in saline environments is urgent for the application of this turfgrass in salt-affected regions. In this study, physiological responses of the Kentucky bluegrass cultivars “Explorer” and “Blue Best” to NaCl treatment, as well as gene expressions related to photosynthesis, ion transport, and ROS degradation, were analyzed. The results showed that the growth of “Explorer” was obviously better compared to “Blue Best” under 400 mM NaCl treatment. “Explorer” exhibited a much stronger photosynthetic capacity than “Blue Best” under NaCl treatment, and the expression of key genes involved in chlorophyll biosynthesis, photosystem II, and the Calvin cycle in “Explorer” was greatly induced by salt treatment. Compared with “Blue Best”, “Explorer” could effectively maintain Na^+^/K^+^ homeostasis in its leaves under NaCl treatment, which can be attributed to upregulated expression of genes, such as *HKT1;5*, *HAK5,* and *SKOR*. The relative membrane permeability and contents of O_2_^−^ and H_2_O_2_ in “Explorer” were significantly lower than those in “Blue Best” under NaCl treatment, and, correspondingly, the activities of SOD and POD in the former were significantly higher than in the latter. Moreover, the expression of genes involved in the biosynthesis of enzymes in the ROS-scavenging system of “Explorer” was immediately upregulated after NaCl treatment. Additionally, free proline and betaine are important organic osmolytes for maintaining hydration status in Kentucky bluegrass under NaCl treatment, as the contents of these metabolites in “Explorer” were significantly higher than in “Blue Best”. This work lays a theoretical basis for the improvement of salt tolerance in Kentucky bluegrass.

## 1. Introduction

In recent decades, soil salinity has been a major threat to turf establishment and management, especially in arid regions, as the cultivation of turfgrasses in these regions highly depends on reclaimed salt-containing water [1,2,3]. Kentucky bluegrass (*Poa pratensis* L.) is widely utilized in lawns and water and soil conservation, as well as slope protection, owing to its eminent traits, such as winter hardiness, a fine texture, a developed rhizome, and a great capacity for ramet generation [2]. This species is also employed in athletic fields like golf courses [3]. Nevertheless, Kentucky bluegrass displays a high sensitivity to soil salinity [4]. Therefore, elucidating the adaptative mechanisms of Kentucky bluegrass with respect to salinity and improving its salt tolerance through genetic approaches are high priorities for the development of the turf industry.

Salinity directly exerts osmotic stress and ion toxicity on plants or initiates secondary stresses (e.g., oxidative stress), ultimately influencing many aspects of biological processes, such as root activity, tissue hydration, photosynthesis, nutrient balance, membrane integrity, and cellular metabolism [1,5]. Plants deal with osmotic stresses mainly by accumulating large quantities of organic or inorganic osmolytes in cells to enhance water influx under salt conditions [1]. For the majority of glycophytes, the biosynthesis of organic metabolites, including amino acids, soluble sugars, and alkaloids, is the most effective way to decrease tissue osmotic potential and maintain hydration status under salt stresses [6,7]. Na^+^ in saline soils is metabolically toxic to plants and disturbs K^+^ homeostasis in plant tissues [8]. It has been found that restricting Na^+^ transport into photosynthetic organs is essential for Poaceae plants [9,10]. The formation rate of reactive oxygen species (ROS) also accelerates substantially under salt stresses, which leads to membrane dysfunction [11]. The activities of key antioxidases in the ROS-scavenging system can increase the degradation of ROS under salt stresses [12,13]. However, the accumulation patterns of organic osmolytes, as well as the strategies for alleviating ion toxicity and oxidative stresses in Kentucky bluegrass under saline conditions, still require further investigation.

Na^+^/K^+^ transporters are employed by plants to alleviate ion toxicity; for instance, the Na^+^/H^+^ antiporter NHX1 mediates the sequestration of Na^+^ into vacuoles, high-affinity K^+^ transporters (HKTs) mediate Na^+^ and/or K^+^ uptake into cells, and shaker-type K^+^ channels, such as AKT1, are responsible for root K^+^ uptake [14,15,16,17]. Many researchers have reported that the expression of genes associated with Na^+^ and K^+^ transport undergoes significant changes after salt treatments, and these changes play significant roles in maintaining Na^+^/K^+^ homeostasis in plants [18,19,20]. It was discovered that plants can also upregulate the expression of genes encoding antioxidases to enhance ROS degradation under salt stresses [13]. Additionally, the expression of many genes regulating the photosynthetic processes in plants has been found to be influenced significantly by salinity [21,22,23]. Nevertheless, the responses of genes involved in these processes in Kentucky bluegrass have not been well-documented. 

To investigate the physiological response of Kentucky bluegrass to salt stress and preliminarily uncover the molecular basis underlying the maintenance of photosynthesis, as well as the alleviation of Na^+^ toxicity and oxidative stress, we compared the growth and photosynthesis of a salt-tolerant cultivar, “Explorer”, and a salt-sensitive cultivar, “Blue Best”, under NaCl treatment; determined the contents of organic osmolytes, as well as Na^+^ and K^+^, in tissues; and analyzed the ROS contents and activities of antioxidases after salt treatment. We also monitored the expression changes in key genes associated with photosynthesis, Na^+^/K^+^ transport, and ROS degradation in both cultivars under NaCl treatment.

## 2. Results

### 2.1. Effects of NaCl Treatment on the Growth and Photosynthesis of Two Kentucky Bluegrass Cultivars

**Figure 1:**
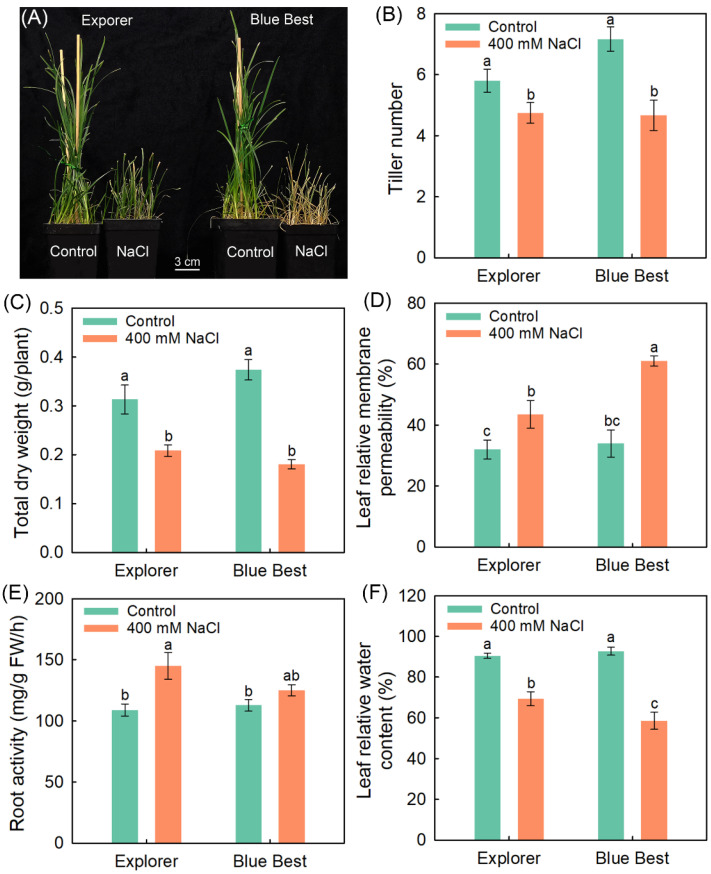
Effects of 400 mM NaCl treatment on growth-related parameters of Kentucky bluegrass cultivars “Explorer” and “Blue Best”. (**A**) Growth photograph, (**B**) tiller number, (**C**) total dry weight, (**D**) leaf relative membrane permeability, (**E**) root activity, and (**F**) leaf relative water content. Data are means (±SDs), *n* = 6. Different letters indicate significant differences as determined using Tukey’s HSD test (*p* < 0.05).

Under 400 mM NaCl treatment, the leaves of “Explorer” remained green, while the leaves of “Blue Best” were visually wilted and yellow (Figure 1A). In “Explorer” and “Blue Best”, the salt treatment significantly reduced the tiller number, total DW, and leaf RWC, while it significantly increased the leaf RMP and root activity (except for the root activity in “Blue Best”) (Figure 1B–F). Notably, under NaCl treatment, the leaf RMP in “Explorer” was significantly lower than that in “Blue Best”, whilst the leaf RWC in the former was notably higher than that in the latter (Figure 1D,E). 

**Figure 2:**
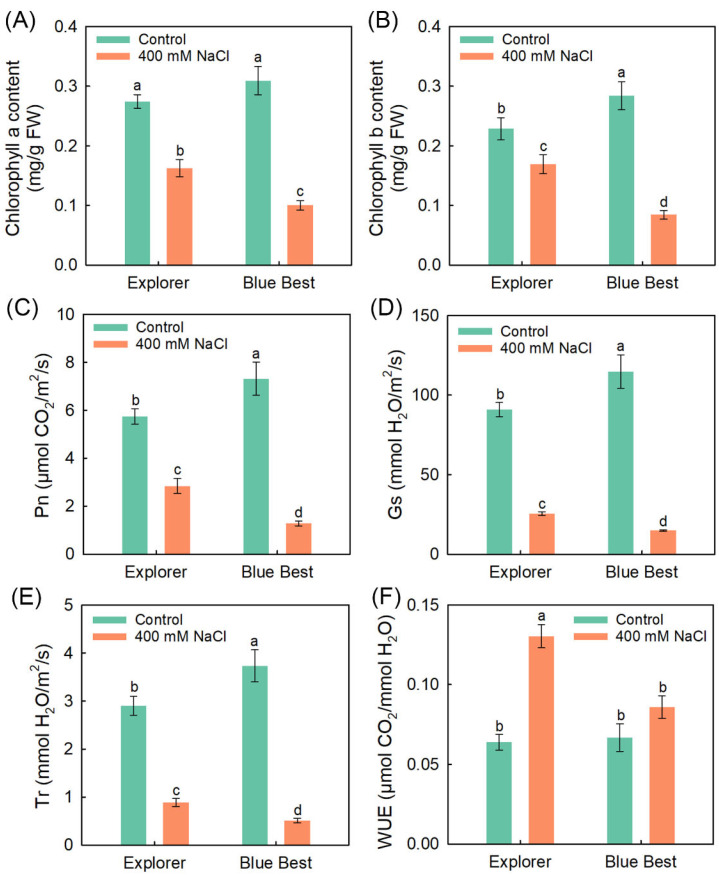
Effects of 400 mM NaCl treatment on photosynthesis-related parameters of Kentucky bluegrass cultivars “Explorer” and “Blue Best”. (**A**) Chlorophyll a content, (**B**) chlorophyll b content, (**C**) net photosynthesis rate (Pn), (**D**) stomatal conductance (Gs), (**E**) transpirational rate (Tr), and (**F**) water-use efficiency (WUE). Data are means (±SDs), *n* = 6. Different letters indicate significant differences as determined using Tukey’s HSD test (*p* < 0.05).

As shown in Figure 2, under the control condition, most photosynthesis-related parameters in “Blue Best”, including chlorophyll b content, Pn, Gs, and Tr, were significantly higher than those in “Explorer”, indicating that the former exhibits a stronger photosynthetic capacity than the latter when grown under normal conditions. The 400 mM NaCl treatment significantly decreased the chlorophyll contents, Pn, Gs, and Tr in both cultivars compared to the control (Figure 2). The WUE in “Explorer” under salt treatment was significantly elevated, while this parameter in “Blue Best” under salt treatment was sustained at the control level (Figure 2F). Moreover, under NaCl treatment, “Explorer” displayed obviously higher chlorophyll contents, Pn, Gs, Tr, and WUE compared to “Blue Best” (Figure 2).

### 2.2. Effects of NaCl Treatment on Tissue Na^+^ and K^+^ Accumulation in Two Kentucky Bluegrass Cultivars

**Figure 3:**
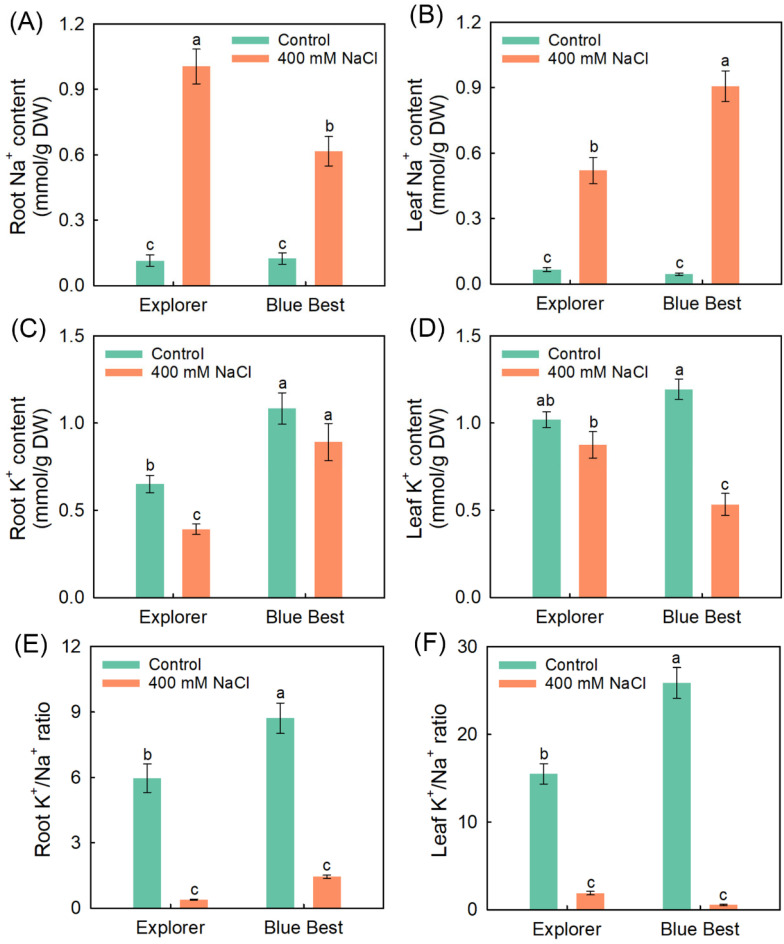
Effects of 400 mM NaCl treatment on tissue K^+^ and Na^+^ contents as well as K^+^/Na^+^ ratio of Kentucky bluegrass cultivars “Explorer” and “Blue Best”. (**A**) Root Na^+^ content, (**B**) leaf Na^+^ content, (**C**) root K^+^ content, (**D**) leaf K^+^ content, (**E**) root K^+^/Na^+^ ratio, and (**F**) leaf K^+^/Na^+^ ratio. Data are means (±SDs), *n* = 6. Different letters indicate significant differences as determined using Tukey’s HSD test (*p* < 0.05).

The tissue Na^+^ contents of both cultivars under NaCl treatment were drastically elevated; furthermore, “Explorer” showed a lower leaf Na^+^ content than “Blue Best” (Figure 3A,B). NaCl treatment did not affect the leaf K^+^ content in “Explorer”, but it significantly decreased this parameter in “Blue Best” (Figure 3C,D). Additionally, the K^+^/Na^+^ ratio in the leaves of “Explorer” was significantly higher than that in “Blue Best” under salt treatment (Figure 3E,F).

### 2.3. Effects of NaCl Treatment on Contents of Organic Osmolytes in the Leaves of Two Kentucky Bluegrass Cultivars

**Figure 4:**
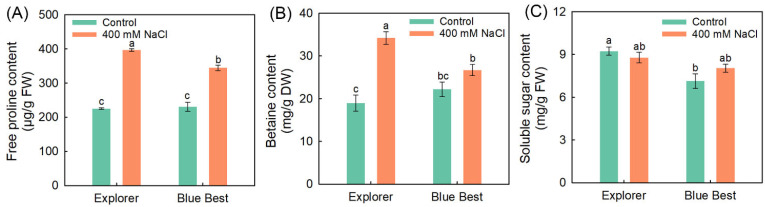
Effects of 400 mM NaCl on leaf organic osmolyte contents of Kentucky bluegrass cultivars “Explorer” and “Blue Best”. (**A**) Free proline content, (**B**) betaine content, and (**C**) soluble sugar content. Data are means (±SDs), *n* = 6. Different letters indicate significant differences as determined using Tukey’s HSD test (*p* < 0.05).

NaCl treatment significantly elevated the free proline and betaine contents in both cultivars, while it did not affect the leaf soluble sugar contents (Figure 4). Under NaCl treatment, no difference in soluble sugar contents between the two cultivars was observed, but the free proline and betaine contents in “Explorer” were significantly higher than those in “Blue Best” (Figure 4).

### 2.4. Effects of NaCl Treatment on ROS Contents and Activities of Antioxidases in the Leaves of Two Kentucky Bluegrass Cultivars

**Figure 5:**
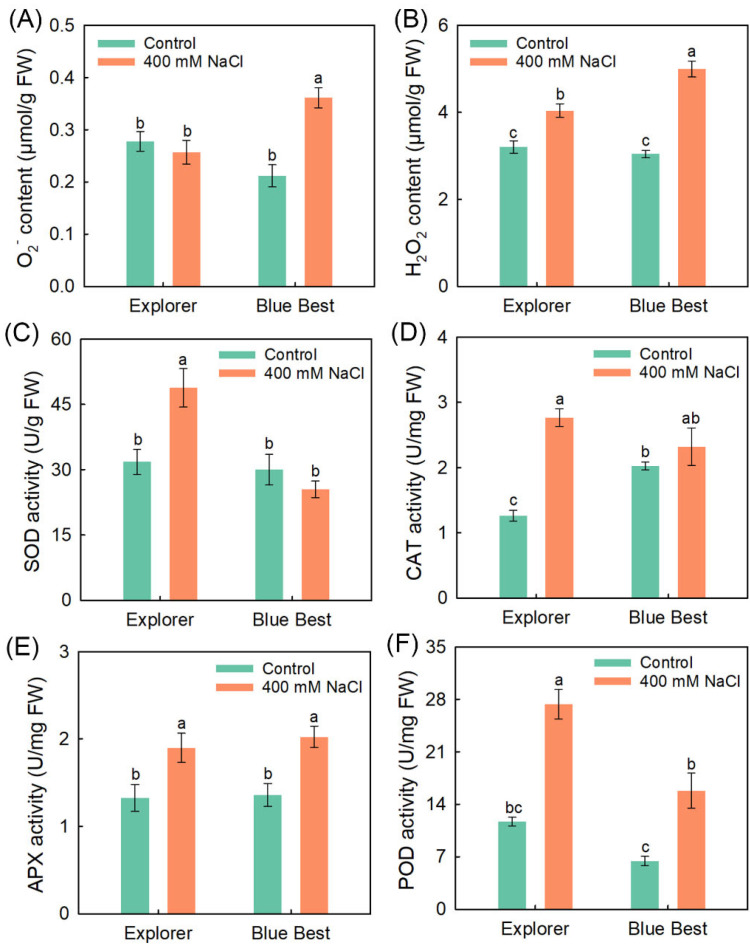
Effects of 400 mM NaCl on contents of O_2_^−^ and H_2_O_2_ as well as activities of antioxidases in leaves of Kentucky bluegrass cultivars “Explorer” and “Blue Best”. (**A**) O_2_^−^ content; (**B**) H_2_O_2_ content; (**C**) superoxide dismutase (SOD) activity; (**D**) catalase (CAT) activity; (**E**) ascorbate peroxidase (APX) activity; (**F**) peroxidase (POD) activity. Data are means (±SDs), *n* = 6. Different letters indicate significant differences as determined using Tukey’s HSD test (*p* < 0.05).

The 400 mM NaCl treatment did not affect the O_2_^−^ content in the leaves of “Explorer”; moreover, the H_2_O_2_ content in “Explorer” was significantly lower than that in “Blue Best” (Figure 5A,B). As shown in Figure 5C,D, the NaCl treatment significantly increased the SOD and CAT activities in “Explorer” but had no effect on their activities in “Blue Best”. Under the NaCl treatment, the APX activity of both cultivars was increased, but no significant difference was observed between “Explorer” and “Blue Best” (Figure 5E). The POD activity in “Explorer” and “Blue Best” under the NaCl treatment was also significantly increased, while the increase in “Blue Best” was smaller than that in “Explorer” (Figure 5F).

### 2.5. Effects of NaCl Treatment on the Expression of Key Genes Associated with Photosynthesis in the Leaves of Two Kentucky Bluegrass Cultivars

**Figure 6:**
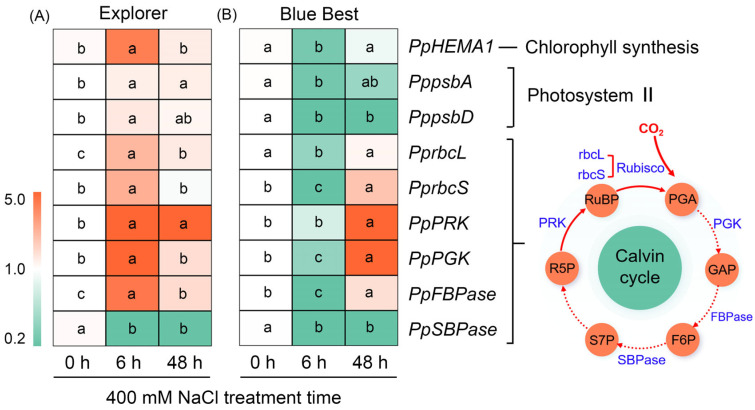
The expression changes of genes related to photosynthesis processes of Kentucky bluegrass cultivars “Explorer” (**A**) and “Blue Best” (**B**) after 400 mM NaCl treatment. Different letters in blocks indicate significant differences as determined using Tukey’s HSD test (*p* < 0.05). HEMA: glutamyl-tRNA reductase, psbA: photosystem II protein D1, psbD: photosystem II protein D2, rbcL: ribulose 1,5-bisphosphate carboxylase/oxygenase large subunit, rbcS: ribulose 1,5-bisphosphate carboxylase/oxygenase small subunit, PRK: phosphoribulokinase, PGK: phosphoglycerate kinase, FBPase: fructose-1,6-bisphosphatase, SBPase: sedoheptulose-1,7-bisphosphatase, RuBP: ribulose-1,5-bisphosphate, PGA: glycerate-3-phosphate, GAP: glyceraldehyde-3-phosphate, F6P: fructose-6-phosphate, S7P: sedoheptulose-7-phosphate, R5P: ribulose-5-phosphate.

We identified nine genes associated with photosynthesis—*HEMA1* (responsible for chlorophyll biosynthesis [24]), *psbA,* and *psbD* (which encode subunits in photosystem II [25]), *rbcL*, *rbcS*, *PRK*, *PGK*, *FBPase*, and *SBPase* (all involved in the Calvin cycle [26])—in the leaves of both Kentucky bluegrass cultivars. The expression of *PpHEMA1*, *PppsbA*, and *PppsbD* in “Explorer” was induced or maintained at high levels after salt treatment; in contrast, these genes in “Blue Best” under salt treatment were substantially downregulated (Figure 6 and Table S2). *PprbcL*, *PprbcS*, *PpPRK*, *PpPGK*, and *PpFBPase* in “Explorer” were immediately upregulated after short-term treatment (Figure 6 and Table S2), whilst these genes (except for *PpPRK*) were downregulated after 6 h of treatment, and some members (such as *PprbcS*, *PpPRK*, *PpPGK*, and *PpFBPase*) showed increasing trends when the treatment time was prolonged to 48 h (Figure 6 and Table S2). In addition, the expression of *PpSBPase* after salt treatment was downregulated in both cultivars (Figure 6 and Table S2).

### 2.6. Effects of NaCl Treatment on the Expression of Key Genes Related to Na^+^ and K^+^ Transport in the Roots of Two Kentucky Bluegrass Cultivars

**Figure 7:**
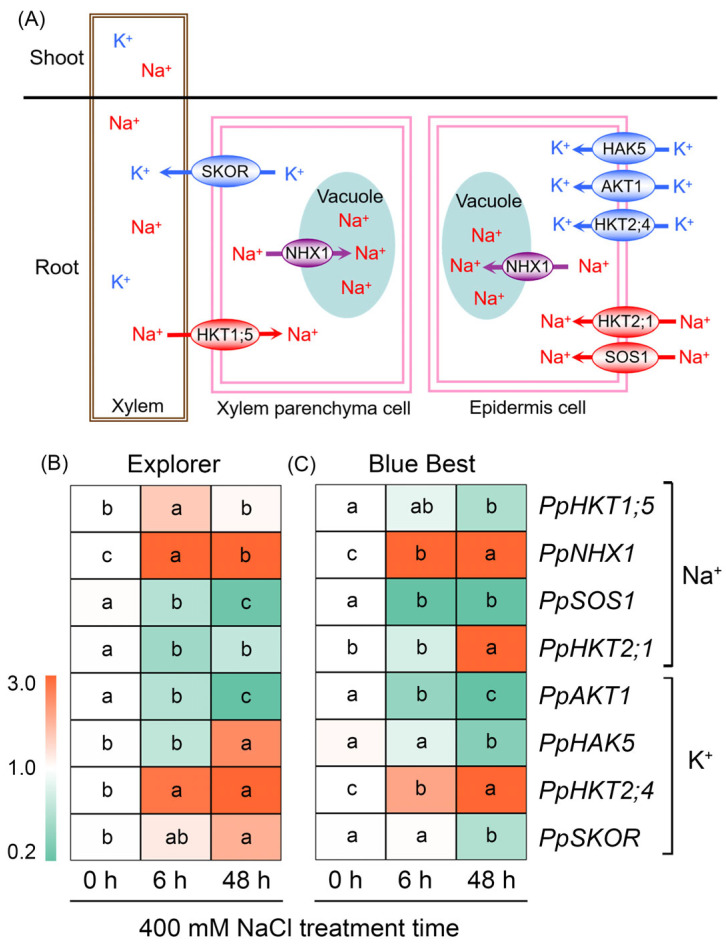
The diagram of proteins involved in Na^+^/K^+^ transport in plants (**A**). The HAK5, AKT1, and HKT2;4 mediate the uptake of K^+^ into the root epidermis cells; the HKT2;1 and SOS1 mediate the uptake of Na^+^ into the root epidermis cells; the NHX1 mediates the transport of Na^+^ into vacuoles; the SKOR mediates the efflux of K^+^ from root xylem parenchyma cells; the HKT1;5 mediates the unloading of Na^+^ from root xylem into parenchyma cells. The expression changes of genes related to Na^+^ and K^+^ transport in Kentucky bluegrass cultivars “Explorer” (**B**) and “Blue Best” (**C**) after 400 mM NaCl treatment. Different letters in blocks indicate significant differences as determined using Tukey’s HSD test (*p* < 0.05). HKTs and HAK5: high-affinity K^+^ transporters, NHX1: tonoplast Na^+^/H^+^ antiporter, SOS1: plasma membrane Na^+^/H^+^ antiporter, AKT1: inwardly rectifying K^+^ channel, SKOR: stelar K^+^ outwardly rectifying channel.

It has been documented that NHX1 mediates the sequestration of Na^+^ into vacuoles and SOS1 mediates the efflux of Na^+^ from roots [27,28]. *PpNHX1* in “Explorer” and “Blue Best” showed an increasing trend, while *PpSOS1* in both cultivars exhibited a declining trend after salt treatment (Figure 7B,C and Table S3). HKTs are thought to govern the uptake of Na^+^ and/or K^+^ into plant cells, among which HKT1;5 is involved in root xylem Na^+^ unloading, and HKT2;1 mediates Na^+^ absorption by roots [29,30]. The expression of *PpHKT1;5* was greatly induced in “Explorer” after short-term treatment but suppressed in “Blue Best” after long-term treatment; the expression of *PpHKT2;1* in “Explorer” was constantly downregulated after salt treatment, while it was upregulated in “Blue Best” after long-term NaCl treatment (Figure 7B,C and Table S3). 

HKT2;4 is thought to mediate the uptake of K^+^ into plant cells [31]. The expression of *PpHKT2;4* was highly inducible by NaCl treatment in both Kentucky bluegrass cultivars (Figure 7B,C and Table S3). Shaker-type potassium channels (including AKT1) and potassium transporters (including HAK5) also mediate K^+^ uptake [32]. As shown in Figure 7 and Table S3, the expression of *PpAKT1* in both cultivars and *PpHAKT5* in “Explorer” was constantly downregulated after NaCl treatment, while *PpHAKT5* in “Blue Best” was inducible by long-term treatment. SKOR mediates the translocation of K^+^ into shoots [33]. Interestingly, *PpSKOR* was substantially downregulated in “Blue Best” but remained stable or was significantly upregulated in “Explorer” after salt treatment (Figure 7B,C and Table S3).

### 2.7. Effects of NaCl Treatment on the Expression of Key Genes Involved in ROS Degradation in the Leaves of Two Kentucky Bluegrass Cultivars

**Figure 8:**
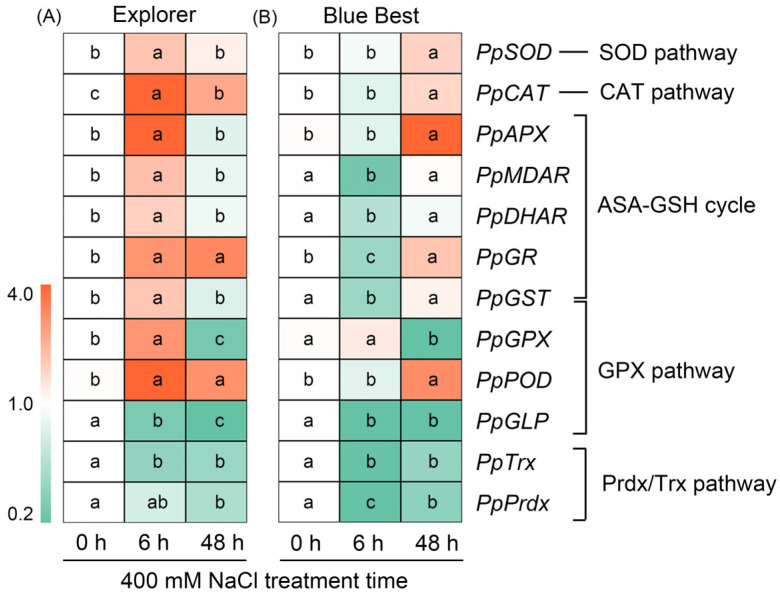
The expression changes of genes related to biosynthesis of key enzymes in the ROS-scavenging system of Kentucky bluegrass cultivars “Explorer” (**A**) and “Blue Best” (**B**) after 400 mM NaCl treatment. Different letters in blocks indicate significant differences as determined using Tukey’s HSD test (*p* < 0.05). SOD: superoxide dismutase, CAT: catalase, APX: ascorbate peroxidase, MDAR: monodehydroascorbate reductase, DHAR: dehydroascorbate reductase, GR: glutathione reductase, GST: glutathione S-transferase, GPX: glutathione peroxidase, POD: peroxidases, GLP: germin-like protein, Trx: thioredoxin, Prdx: peroxiredoxin.

The ROS-scavenging system in plants constitutes the SOD pathway, GPX pathway, CAT pathway, Prdx/Trx pathway, and AsA-GSH cycle [23]. The expression of *PpSOD* and *PpCAT* (involved in the SOD pathway and CAT pathway, respectively) in “Explorer” was upregulated after salt treatment, but in “Blue Best” they were downregulated after short-term treatment (Figure 8 and Table S4). Interestingly, after 6 h of treatment, the expression of *PpAPX*, *PpMDAR*, *PpDHAR*, *PpGr*, and *PpGST* in “Explorer” was significantly upregulated, while most of these genes in “Blue Best” were downregulated (Figure 8 and Table S4). It was found that the expression of *PpGPX* and *PpPOD* (involved in the GPX pathway) in “Explorer” was substantially upregulated (over 3-fold) after NaCl treatment, while the expression of *PpGLP* (also involved in the GPX pathway) in both cultivars was significantly downregulated after salt treatment. Furthermore, *PpTrx* and *PpPrdx* (both involved in the Prdx/Trx pathway) in both cultivars were also substantially downregulated under salt treatment (Figure 8 and Table S4).

## 3. Discussion

### 3.1. Maintaining a High Photosynthetic Performance by Upregulating the Expression of Photosynthesis-Related Genes Is Important for Salt Tolerance in Kentucky Bluegrass

Although Kentucky bluegrass is a typical glycophyte that is more sensitive to salinity than many other turfgrasses, this species is thought to have high plasticity in terms of salt adaptation [3,34]. We previously found that the Kentucky bluegrass cultivar “Explorer” is a relatively salt-tolerant species and “Blue Best” is a sensitive one [35]. Here, “Explorer” also exhibited a much stronger salt tolerance than “Blue Best”, as evidenced by the fact that the shoot growth and leaf hydration of the former were significantly better than those of the latter under 400 mM NaCl treatment for 15 d (Figure 1). Thus, the elucidation of the prominent traits possessed by “Explorer” relative to “Blue Best” under salt stress would provide a theoretical basis for improving the salt tolerance of Kentucky bluegrass using genetic approaches.

The net photosynthesis rate (Pn) is used to represent the photosynthetic capacity of plants, and it has been found that the Pn in most glycophytes (and even some halophytes) is inhibited greatly under saline conditions [36,37,38]. Although the Pn in the two Kentucky bluegrass cultivars significantly decreased under salt treatment, the decrease in “Explorer” was much smaller than that in “Blue Best” (Figure 2C). Given that “Explorer” exhibits a stronger salt tolerance than “Blue Best” (Figure 1), the ability to sustain a relatively high photosynthetic capacity is essential for Kentucky bluegrass to adapt to salinity. The stomatal pores constitute the only means for CO_2_ to enter the photosynthesis organs [39]. Notably, the stomatal conductance in “Explorer” under salt treatment was significantly higher than that in “Blue Best” (Figure 2D), suggesting that the maintenance of stomatal opening to ensure CO_2_ acquisition is also closely related to the salt tolerance of Kentucky bluegrass.

The biosynthesis process of chlorophyll is hindered in many plants under salt stress [5,40]. In this study, it was observable that the leaves of “Explorer” remained green but most tillers of “Blue Best” turned yellow under the 400 mM NaCl treatment (Figure 1A). Correspondingly, the chlorophyll a and b contents in “Blue Best” sharply declined after the salt treatment (Figure 2A,B). HEMA is a rate-limiting enzyme that catalyzes chlorophyll biosynthesis, and *HEMA1* in rice (*Oryza sativa*) is substantially upregulated under salt treatments [24,40]. In this study, *PpHEMA1* under NaCl treatment showed upregulated expression in “Explorer” but downregulated expression in “Blue Best” (Figure 6). These results suggest that “Explorer” should possess a stronger ability to maintain chlorophyll biosynthesis than “Blue Best”.

The expression of genes encoding components in PSII, such as *psbA*, *psbD*, and *psbQ* in sweet sorghum and black locust, has been found to substantially decline after salt treatments [22,25]. In this study, *PppsbA* and *PppsbD* showed opposing expression patterns in “Explorer” (significantly upregulated) and “Blue Best” (significantly downregulated) after NaCl treatment (Figure 6), indicating that the upregulation of *psbA* and *psbD* should also play vital roles in maintaining photosynthetic capacity in Kentucky bluegrass under saline conditions. We also analyzed the expression of key genes in the Calvin cycle and found that, except for *PpSBPase*, the expression of other determined genes in “Explorer”—including *PprbcL*, *PprbcS*, *PpPRK*, *PpPGK*, and *PpFBPase*—was immediately upregulated after short-term salt treatment and maintained at high levels when the treatment time was prolonged to 48 h; in contrast, most of these genes in “Blue Best” showed suppressed expression under salt treatment (Figure 6). Therefore, the rapid upregulation of genes in the Calvin cycle could be another reason why “Explorer” displays relatively high salt adaptation. 

### 3.2. Restricting Na^+^ Overaccumulation and Maintaining K^+^ Homeostasis in Leaves Play Essential Roles in Kentucky Bluegrass’s Adaptation to Saline Environments

There are diverse strategies for plants to cope with Na^+^ toxicity; for example, plants with succulent leaves can accumulate Na^+^ into developed vacuoles, and some halophytes can export Na^+^ through salt-secreting organs on the leaf surface [41,42]. However, for most plant species within the Poaceae family, restricting the transport of Na^+^ into leaves is more important for alleviating Na^+^ toxicity [30,43]. In this study, “Explorer” under NaCl treatment showed a significantly lower leaf Na^+^ content compared to “Blue Best” (Figure 3A,B). Furthermore, the Na^+^ content in the roots of “Explorer” was approximately 2-fold higher than that in the leaves, while this parameter in the roots of “Blue Best” (approximately 0.6 mmol/g DW) was lower than that in the leaves (approximately 0.9 mmol/g DW) under NaCl treatment (Figure 2A,B). Therefore, the restriction of Na^+^ overaccumulation in leaves is critical for the salt tolerance of Kentucky bluegrass.

The root xylem’s unloading of Na^+^ is particularly important for restricting Na^+^ accumulation in shoots, and HKT1;5 has been proven to mediate this process [44,45]. *PpHKT1;5* was significantly upregulated in the roots of “Explorer”, while in “Blue Best” it was downregulated, after salt treatment (Figure 7B,C). Therefore, the enhanced expression of *PpHKT1;5* may be closely associated with the restriction of Na^+^ overaccumulation in the leaves of Kentucky bluegrass under salt stress. In addition, HKT2;1 has been discovered to mediate roots’ Na^+^ absorption, and both *TaHKT2;1* in wheat (*Triticum aestivum*) and *OsHKT2;1* in rice are upregulated under salt stress [29,46]. Interestingly, *PpHKT2;1* in “Explorer” exhibited substantially decreased expression after NaCl treatment, while this gene in “Blue Best” was upregulated after treatment for 48 h (Figure 7B,C). Therefore, the downregulation of *HKT2;1* may also be essential for alleviating Na^+^ toxicity in Kentucky bluegrass under salt stress. 

Na^+^ in saline soils would disrupt the K^+^ homeostasis in most plants [41]. Interestingly, the leaf K^+^ content under NaCl treatment in “Explorer” remained at a relatively high level, while this parameter in “Blue Best” sharply declined after salt treatment (Figure 3C,D). A K^+^/Na^+^ ratio < 1 in plant tissues is considered to exert significant disturbances on the metabolism in plants [1]. We found that the leaf K^+^/Na^+^ ratio in “Explorer” was approximately 2, while it was less than 0.6 in “Blue Best”, under salt stress (Figure 3F). AKT1 and HAK5 constitute important pathways for K^+^ absorption in roots, and *AKT1* has been reported to be upregulated in some xerophytic species under salt stress [19,32]. In contrast, the expression of *PpAKT1* in both Kentucky bluegrass cultivars was significantly downregulated after NaCl treatment (Figure 7B,C). Notably, *PpHAK5* in the roots of “Blue Best” was also downregulated after NaCl treatment, while this gene in the roots of “Explorer” was significantly upregulated under salt treatment (Figure 7B,C). Therefore, the increased expression of *HAK5* might be indispensable for the acquisition of K^+^ by Kentucky bluegrass under saline conditions. Additionally, *PpSKOR* (responsible for the transport of K^+^ into shoots) in “Blue Best” remained stable or even significantly declined after NaCl treatment, whilst this gene in “Explorer” displayed significantly upregulated expression after NaCl treatment (Figure 7B,C), which may be a primary reason why this cultivar could maintain K^+^ homeostasis in leaves under salt treatment (Figure 3D). All of these results suggest that improving K^+^ uptake and translocation ability would be a promising way to enhance the salt tolerance of Kentucky bluegrass. 

### 3.3. Enhancing the Ability to Degrade ROS Is Crucial for Kentucky Bluegrass to Cope with Salt Stress

ROS are continuously produced during the metabolic processes of plants; however, their production is elevated under various stress conditions, further exerting oxidative stress on the plants [11]. The ROS content in terms of O_2_^−^ and H_2_O_2_ in “Explorer” under NaCl treatment was much lower than that in “Blue Best” (Figure 5A,B). Correspondingly, the leaf RMP in “Explorer” was substantially lower than that in “Blue Best” after salt treatment (Figure 1D). These results suggest that “Explorer” has a stronger ability to degrade ROS than “Blue Best” under salt stress. The ROS-scavenging system, consisting of many antioxidases, is the most effective way to relieve the damage to cell membranes and organelles caused by ROS [23]. The activities of antioxidases (especially SOD and POD) in “Explorer” substantially increased after NaCl treatment (Figure 5), indicating that the enhanced activities of antioxidases could be particularly important for alleviating oxidative stress in Kentucky bluegrass. 

The increased biosynthesis of antioxidases could also enhance the capacity to degrade ROS in plants [23]. Our results showed that the genes encoding key antioxidases involved the SOD, CAT, and GPX pathways, and the ASA-GSH cycle of ROS-scavenging systems in the leaves of “Explorer”, were immediately upregulated after NaCl treatment, while the expression of most genes in this system was stable or downregulated in the leaves of “Blue Best” (Figure 8), suggesting that the rapid upregulation of genes encoding antioxidases could also contribute to the adaptation of Kentucky bluegrass to salt stress.

### 3.4. The Increased Accumulation of Organic Osmolytes Plays Essential Roles in the Osmotic Adjustment of Kentucky Bluegrass

K^+^ is the most important inorganic osmolyte in plants [47]. However, it was observed that the K^+^ contents in the tissues of the Kentucky bluegrass cultivars did not increase under NaCl treatment (Figure 3D). In contrast, the contents of organic osmolytes in the leaves of the Kentucky bluegrass cultivars were substantially elevated after NaCl treatment (Figure 5). The leaf hydration status in “Explorer” under NaCl treatment was substantially better than that in “Blue Best” (Figure 1F). Correspondingly, the contents of free proline and betaine in “Explorer” were obviously higher than those in “Blue Best” (Figure 5). Therefore, the increased biosynthesis of proline and betaine may be important for Kentucky bluegrass to maintain hydration status under saline conditions.

In addition to serving as an organic osmolyte, proline in plants is also a non-enzymatic antioxidant; meanwhile, it can increase the activities of antioxidases, such as CAT [48]. Considering that “Explorer” displayed a much stronger ability to degrade ROS than “Blue Best”, the increased accumulation of proline in leaves may also be important for alleviating oxidative stress under saline conditions. Taken together, the large-scale biosynthesis of organic osmolytes, such as free proline and betaine, may play an essential role in the salt tolerance of Kentucky bluegrass.

## 4. Materials and Methods

### 4.1. Plant Materials and Growth Conditions

Seeds of the Kentucky bluegrass cultivars “Explorer” and “Blue Best” were first placed in Petri dishes for germination, and then the seedlings were transferred into 0.4 L plastic pots containing silica sand (8 seedlings/pot) and irrigated with Hoagland solution [43]. All seedlings were constantly cultured in a growth chamber with 16 h/8 h light/dark at 25/20 °C, 60% relative humidity, and ~600 μmol·m^−2^·s^−1^ of illumination intensity. 

After 2 months, uniform seedlings of both cultivars were selected. Our previous study evaluated the salt tolerance of 30 Kentucky bluegrass cultivars under 400 mM NaCl treatment, and we found that detrimental effects of 400 mM NaCl treatment on the growth of “Blue Best” were more severe than those on “Explorer” [35]. Therefore, to analyze the physiological responses of these two cultivars to salt stress, the seedlings were exposed to Hoagland solution containing 400 mM NaCl, and seedlings that we continued to irrigate with Hoagland solution were assigned as the control. All solutions were renewed every 2 d. After 15 d, all seedlings in one pot were harvested and assigned as one replicate. Six replicates were used for all measurements (*n* = 6).

To monitor the expression of genes related to photosynthesis, Na^+^ and K^+^ transport, and ROS degradation in Kentucky bluegrass after salt treatment, uniform seedlings were exposed to 400 mM NaCl treatment solution for 0 (control), 6, and 48 h. Three replicates were used for the gene expression analysis (*n* = 3).

### 4.2. Determination of Growth-Related Parameters

The tiller numbers of the seedlings were counted first, and then the whole plant was placed in an oven at 80 °C for 3 d, after which the total dry weight (DW) was measured. Finally, the leaves’ relative water content (RWC) was calculated [5].

For determination of the leaves’ relative membrane permeability (RMP), all leaves of the seedlings were collected, immersed in deionized water, vacuum-filtered 3 times, and shaken at 25 °C. After 2 h, the electrolyte leakage was determined with a conductivity meter. After incubation at 100 °C for 2 h, the total electrolytes of the leaves were determined. The RMP was calculated as described by Cui et al. [5].

All roots of the seedlings were collected, immersed in 0.4% TTC solution at 37 °C in the dark, and then sealed in methyl alcohol for 3 h. Finally, the absorbance was recorded at 485 nm using a UV spectrophotometer (UV-2102C, Unico Instrument Co., Ltd., Shanghai, China) to calculate the root activity [49].

### 4.3. Determination of Photosynthesis-Related Parameters

The net photosynthesis rate (Pn), stomatal conductance (Gs), transpiration rate (Tr), and water-use efficiency (WUE) were measured in the growth chamber between 3 h and 5.5 h after the start of photoperiod using the LI-6800 Portable Photosynthesis System [5].

Fresh leaf samples weighing approximately 1 g were collected and then crushed with a mixed solution of 80% acetone and 95% ethanol. Then, the absorbances at 645 nm and 663 nm were determined to calculate the chlorophyll (Chl) a and Chl b contents [50].

### 4.4. Determination of Tissue Na^+^ and K^+^ Contents

Thoroughly dried root and leaf samples weighing approximately 0.1 g were immersed in 100 mM glacial acetic acid at 90 °C. After filtering, the solutions were collected to determine the Na^+^ and K^+^ contents using a flame spectrophotometer (Model 410 Flame; Sherwood Scientific, Ltd., Cambridge, UK) [51]. The tissue K^+^/Na^+^ ratio was calculated as K^+^ content/Na^+^ content.

### 4.5. Determination of the Contents of Free Proline, Betaine, and Soluble Sugars in Leaves

Fresh leaf samples weighing 0.5 g were homogenized with 3% sulfosalicylic acid at 100 °C. The supernatant was reacted with the mixed solution of 2.5% acid–ninhydrin and glacial acetic acid at 100 °C for 1 h. The free proline was leached with toluene, and then the absorbance at 520 nm was measured to calculate the free proline content [52].

The betaine content in 0.1 g dried leaf samples was determined using a Reinecke Salt Kit (Comin Biotechnology, Co., Ltd., Suzhou, China) [53].

Fresh leaf samples weighing 0.5 g were homogenized with 80% ethanol in a boiling water bath for 1 h, and then the soluble sugar content was determined via the classic anthrone colorimetric method [54].

### 4.6. Determination of the Contents of O_2_^−^ and H_2_O_2_ in Leaves and the Activities of SOD, POD, CAT, and APX

The O_2_^−^ content in fresh leaf samples was determined with an O_2_^−^ content detection kit (Solarbio, Beijing, China), and the H_2_O_2_ content in fresh leaf samples was determined with a H_2_O_2_ content detection kit (Jiangcheng, Nanjing, China) [55].

The activities of antioxidases, including superoxide dismutase (SOD), peroxidase (POD), catalase (CAT), and ascorbate peroxidase (APX), were determined using the enzyme activity assay kits (Solarbio, Beijing, China) [56].

### 4.7. Real-Time Quantitative PCR Analysis

The leaves and roots were transiently frozen, and then the total RNA was extracted with the TRIzol reagent (Tiangen, Beijing, China). The gene-specific primer pairs with product lengths of 180–220 bp were designed using Premier Primer 5.0 (Table S1). After removal of genomic DNA, RNA samples were converted into cDNA [57]. The cDNA samples were used as templates for qRT-PCR using a Real-Time PCR Thermocycler (QuantStudio7 Flex, Thermo Fisher Scientific, Waltham, MA, USA) [58]. 

### 4.8. Statistical Analysis

The data were subjected to one-way analysis of variance (Tukey’s HSD, *p* < 0.05) using SPSS 19.0 (IMB Corp, Armonk, NY, USA). All histograms were drawn with SigmaPlot 14.0, and all heatmaps were drawn using the Multiple Experiment Viewer 4.9.0 (J. Craig Venter Institute, La Jolla, CA, USA).

## 5. Conclusions

In conclusion, this study obtained the following findings: (I) the increased expression of genes associated with chlorophyll biosynthesis, components in PSII, and the Calvin cycle contributes to the maintenance of photosynthesis of Kentucky bluegrass under salt stress; (II) the restriction of Na^+^ overaccumulation in leaves to maintain K^+^ homeostasis by upregulating the expression of genes involved in Na^+^/K^+^ transport (such as *HKT1;5*, *HAK5*, and *SKOR*) plays a key role in Kentucky bluegrass’s adaptation to saline environments; (III) the enhanced activities of antioxidases, such as SOD and POD, along with the increased expression of genes involved in the biosynthesis of enzymes in the ROS-scavenging system, are crucial for ROS degradation in Kentucky bluegrass under saline conditions; and (IV) the significant accumulation of free proline and betaine should be important for maintaining the hydration status of Kentucky bluegrass under salt stress. 

## Data Availability

The data that support the findings of this study are available from the corresponding author upon reasonable request.

## Supplementary Materials

The following supporting information can be downloaded at: https://www.mdpi.com/article/10.3390/plants13152107/s1, Table S1: The primers for qRT-PCR analysis; Table S2: The relative expression level of genes related to photosynthesis in Kentucky bluegrass cultivars “Explorer” and “Blue Best” under 400 mM NaCl for 0 h (control), 6 h, or 48 h; Table S3: The relative expression level of genes related to Na^+^ and K^+^ transport in roots of Kentucky bluegrass cultivars “Explorer” and “Blue Best” under 400 mM NaCl for 0 h (control), 6 h, or 48 h; Table S4: The relative expression level of genes related to the ROS-scavenging system in leaves of Kentucky bluegrass cultivars “Explorer” and “Blue Best” under 400 mM NaCl for 0 h (control), 6 h, or 48 h.
