# Supplementary material for: Comparative Physiological and Gene Expression Analyses Reveal Mechanisms Involved in Maintaining Photosynthesis Capacity, Alleviating Ion Toxicity and Oxidative Stress of Kentucky Bluegrass under NaCl Treatment
Source: Plants (Basel). 2024 Jul 30;13(15):2107. doi: 10.3390/plants13152107 (PMC11313982; doi:10.3390/plants13152107)
Supplement: Supplementary file 1 [file plants-13-02107-s001.zip › plants-3117955-supplementary.pdf]

Table S1 The primers for qRT-PCR analysis.

Genes	Forward primers (5′-3′)	Reserve primers (5′-3′)
**Ion transports**		
PpHKT1;5	GCGATGCTCCTTTCTTCTTG	GCTCCTTACCCTCCTTATGCT
PpNHX1	GCTAGGATTGGTTCTGGTTGG	CTTATTTTCTCGGCGGGC
PpSOS1	TCTCGCTCCTTTTCCACC	CTGACAATAACCTCTTCTCCC
PpHKT2;1	AGAAGTTGAGTGGATCGTTTCT	GGCCTTTTCACCGCTATG
PpAKT1	CCAAACCCAAAGCAAACG	GATAATCCCCATAACCCACAG
PpHAK5	GCTGGGTCACGACACTACA	AGACATCACACCAAGGAGG
PpHKT2;4	ATCACTCTCCAGCGACCCT	CCCCACATTCCCATATGC
PpKOR	GTGGGCGAGTCAAACAACT	CCATAAAAAGCGGCGGTAT
**Photosynthesis-related genes**		
PpHEMA1	TGGGCTTGACTCTTTGGTTC	GCTGTGATTGCATCCTTGAAC
PppsbA	GAAAATGAATCCGCTAACGAG	CAGCCAAGAAGAAGTGTAAAGA
PppsbD	ACCGGAAGCCCAAGGAGAT	ATTGAACAGACCGAGCAAG
PprbcL	GAGGGTATTCGTGCGTGGA	GCTTTAGTTTCTGTTTGTGGTGA
PprbcS	GGCTTGAAGGCGATGAAG	TGGAGGAGGTGAAGAAGGAGTA
PpPRK	CTTGACCCCCCAGAACTCAT	GCTCTCTCACACGCTCATCG
PpPGK	GTCATCCTCGCCAGCCATCT	TCATTCGCCATCACAACCTC
PpFBPase	GTACTTGACATCATGCCCACA	CCACCTTCTCCACTTCCTCC
PpSBPase	GGTGTGTGGCCTGGAGATAAG	GGAGGAGGAATTCGTGGGT
**Genes related to ROS-scavenging system**		
PpSOD	CAGGAAATGCTGGTGGGAG	GGGACAATAGAACTGAAGGC
PpCAT	CCTCTTATCGGCAAGGGTT	CGACAGTGCGTGGAATGA
PpAPX	CCATTTCCCCAAACACGC	AGAGACCCCAGATATCCCGC
PpMDAR	CATCCAGGAAAGCAAGAAAGA	GATACAGCCGCAAAAACCC
PpDHAR	CCAAGCCAACCAGGGAGAA	GAAAGGGACTGATAAGACACGA
PpGR	CCAGACATCCCAGGAATAGAGC	GAAAATGCCAGCAAACTCCA
PpGST	CAATGGCGTCGGAGAAGA	CGTAGGTGTAGGGCAGGTTC
PpGPX	AAGTGCTGGAGGATTTTTGGG	TGGAATGGTGAAGTCGTTGGT
PpPOD	CACGCTAATCTCCAAGTTCG	TGTCGTTGTAGATGTGGTCG
PpGLP	CCTTCTCAGCTGAAACCAGAGG	ACATGACATGCACACGCGC
PpTrx	CCAGAGAAAGATCGAGGAGCA	TCACAGCCAAGCGGACAAC
PpPrdx	GCTGATGGATCGGGAACAT	AACCGCCTCGAACGAAGAC
**Reference gene (glyceraldehyde-3-phosphate dehydrogenase)**		
PpGAPDH	TGGTATTGTTGAGGGTCTGATG	CAGTGCTGCTTGGAATGATG

Table S2 The relative expression level of genes related to photosynthesis in Kentucky bluegrass cultivars "Explorer" and "Blue Best" under 400 mM NaCl for 0 h (Control), 6 h or 48 h. Data are means (± SE), n = 3. Different letters indicate significant differences at $P < 0.05$ (Tukey's HSD test).

Genes	Explorer			Blue Best		
	**0 h**	**6 h**	**48 h**	**0 h**	**6 h**	**48 h**
PpHEMA1	1.13 ± 0.07b	4.34 ± 0.42a	1.51 ± 0.11b	1.08 ± 0.02a	0.28 ± 0.01b	0.91 ± 0.03a
PppsbA	1.01 ± 0.10b	1.42 ± 0.05a	1.47 ± 0.05a	1.05 ± 0.04a	0.28 ± 0.04b	0.47 ± 0.01ab
PppsbD	1.03 ± 0.15b	1.58 ± 0.03a	1.30 ± 0.13ab	1.01 ± 0.06a	0.14 ± 0.01b	0.20 ± 0.00b
PprbcL	1.00 ± 0.06c	2.85 ± 0.19a	1.57 ± 0.36b	1.00 ± 0.10a	0.45 ± 0.03b	1.28 ± 0.25a
PprbcS	1.00 ± 0.05b	3.09 ± 0.34a	0.96 ± 0.12b	1.00 ± 0.02b	0.09 ± 0.00c	2.55 ± 0.25a
PpPRK	1.02 ± 0.06b	15.22 ± 1.16a	11.58 ± 0.16a	1.02 ± 0.04b	0.78 ± 0.01b	5.47 ± 0.19a
PpPGK	1.01 ± 0.10b	5.74 ± 0.72a	1.88 ± 0.02b	1.01 ± 0.02b	0.43 ± 0.03c	9.03 ± 0.40a
PpFBPase	1.03 ± 0.16c	4.57 ± 0.45a	1.93 ± 0.15b	1.02 ± 0.01b	0.05 ± 0.00c	1.87 ± 0.04a
PpSBPase	1.13 ± 0.04a	0.20 ± 0.03b	0.21 ± 0.03b	1.04 ± 0.08a	0.12 ± 0.02b	0.12 ± 0.00b

Table S3 The relative expression level of genes related to Na^+ and K^+ transport in roots of Kentucky bluegrass cultivars "Explorer" and "Blue Best" under 400 mM NaCl for 0 h (Control), 6 h or 48 h. Data are means (± SE), $n = 3$. Different letters indicate significant differences at $P < 0.05$ (Tukey's HSD test).

**Genes**	**Explorer**			**Blue Best**		
	**0 h**	**6 h**	**48 h**	**0 h**	**6 h**	**48 h**
PpHKT1;5	1.00 ± 0.08b	1.69 ± 0.08a	1.10 ± 0.08b	1.00 ± 0.07a	0.88 ± 0.00ab	0.57 ± 0.09b
PpNHX1	1.01 ± 0.11c	34.64 ± 4.65a	14.52 ± 1.42b	1.00 ± 0.14c	16.54 ± 0.56b	63.76 ± 6.36a
PpSOS1	1.00 ± 0.22a	0.61 ± 0.01b	0.24 ± 0.02c	1.00 ± 0.28a	0.08 ± 0.00b	0.13 ± 0.01b
PpHKT2;1	1.02 ± 0.14a	0.50 ± 0.04b	0.69 ± 0.05b	1.00 ± 0.17b	0.78 ± 0.23b	5.84 ± 0.38a
PpAKT1	1.00 ± 0.02a	0.61 ± 0.02b	0.11 ± 0.01c	1.01 ± 0.02a	0.45 ± 0.02b	0.09 ± 0.00c
PpHAK5	1.02 ± 0.10b	0.68 ± 0.17b	2.52 ± 0.07a	1.10 ± 0.15a	0.85 ± 0.10a	0.37 ± 0.04b
PpHKT2;4	1.00 ± 0.04b	1.11 ± 0.08b	3.31 ± 0.01a	1.01 ± 0.04b	1.18 ± 0.12b	6.44 ± 1.2a
PpKOR	1.02 ± 0.04b	1.27 ± 0.15ab	2.04 ± 0.16a	1.02 ± 0.05a	1.04 ± 0.02a	0.59 ± 0.04b

Table S4 The relative expression level of genes related to the ROS-scavenging system in leaves Kentucky bluegrass cultivars "Explorer" and "Blue Best" under 400 mM NaCl for 0 h (Control), 6 h or 48 h. Data are means (± SE), $n = 3$. Different letters indicate significant differences at $P < 0.05$ (Tukey's HSD test).

Genes	Explorer			Blue Best		
	**0 h**	**6 h**	**48 h**	**0 h**	**6 h**	**48 h**
PpSOD	1.00 ± 0.02b	2.15 ± 0.04a	1.31 ± 0.01b	1.03 ± 0.04b	0.94 ± 0.05b	1.89 ± 0.03a
PpCAT	1.02 ± 0.01c	4.92 ± 0.60a	2.70 ± 0.16b	1.03 ± 0.02b	0.85 ± 0.04b	1.81 ± 0.08a
PpAPX	1.02 ± 0.10b	4.14 ± 0.24a	0.83 ± 0.05b	1.08 ± 0.14b	0.84 ± 0.06b	6.18 ± 0.67a
PpMDAR	1.00 ± 0.00ab	1.26 ± 0.05a	0.78 ± 0.01b	1.03 ± 0.02a	0.28 ± 0.00b	1.07 ± 0.01a
PpDHAR	1.01 ± 0.03b	1.93 ± 0.16a	0.85 ± 0.02b	1.00 ± 0.00a	0.61 ± 0.01b	0.94 ± 0.05a
PpGR	1.00 ± 0.02b	3.10 ± 0.05a	3.33 ± 0.07a	1.00 ± 0.03b	0.49 ± 0.00c	2.15 ± 0.25a
PpGST	1.01 ± 0.02a	1.31 ± 0.03a	0.50 ± 0.00b	1.01 ± 0.03a	0.48 ± 0.00b	1.28 ± 0.10a
PpGPX	1.02 ± 0.11b	3.07 ± 0.12a	0.29 ± 0.01c	1.05 ± 0.01a	1.42 ± 0.29a	0.14 ± 0.02b
PpPOD	1.05 ± 0.02b	4.81 ± 0.59a	3.18 ± 0.07a	1.03 ± 0.05b	0.85 ± 0.04b	3.23 ± 0.14a
PpGLP	1.00 ± 0.04a	0.31 ± 0.00b	0.04 ± 0.00c	1.02 ± 0.01a	0.04 ± 0.00b	0.05 ± 0.00b
PpTrx	1.00 ± 0.01a	0.43 ± 0.00b	0.47 ± 0.02b	1.00 ± 0.02a	0.21 ± 0.00b	0.44 ± 0.00b
PpPrdx	1.00 ± 0.01a	0.77 ± 0.01ab	0.57 ± 0.01b	1.00 ± 0.02a	0.19 ± 0.00c	0.41 ± 0.01b
